# Anti-infective and cytotoxic properties of *Bupleurum marginatum*

**DOI:** 10.1186/1749-8546-9-4

**Published:** 2014-01-17

**Authors:** Mohamed L Ashour, Mahmoud Z El-Readi, Razan Hamoud, Safaa Y Eid, Sherweit H El Ahmady, Endalkachew Nibret, Florian Herrmann, Mahmoud Youns, Ahmed Tahrani, Dorothea Kaufmann, Michael Wink

**Affiliations:** 1 Institute of Pharmacy and Molecular Biotechnology, Heidelberg University, Im Neuenheimer Feld 364, 69120 Heidelberg, Germany; 2 Department of Pharmacognosy, Faculty of Pharmacy, Ain Shams University, Cairo, Egypt; 3 Department of Biochemistry, Faculty of Pharmacy, Al-Azhar University, Assiut, Egypt; 4 Department of Functional Genome Analysis, German Cancer Research Center (DKFZ), Heidelberg, Germany; 5 Department of Biochemistry and Molecular Biology, Faculty of Pharmacy, Helwan University, Cairo, Egypt

## Abstract

**Background:**

*Bupleurum marginatum* Wall. ex DC (Apiaceae) is a perennial herb widely used in traditional Chinese and Kampo medicine for the treatment of various infectious diseases. The biological activities of *B. marginatum* have not been fully investigated. This study aims to investigate the antitrypanosomal, antimicrobial and antiviral activities of methanol (ME) and dichloromethane (DCM) extracts of *B. marginatum* aerial parts and the ability of both extracts to inhibit the growth of different cancer cell lines.

**Methods:**

Phytochemical characterization of the extracts was performed by LC-MS profiling. The antitrypanosomal activity was evaluated using the resazurin method. The antimicrobial activity was assessed using agar diffusion and microdilution methods, and the minimum inhibitory concentration (MIC) values were determined. The antiviral activity was determined for 6.25, 12.5, and 50 μg/mL doses using a plaque reduction assay. Cytotoxicity was investigated in eight cancer cell lines (Caco-2, CCL-81, CCRF-CEM, COS-7, HL-60, MIA PaCa-2, MCF-7, and PANC-1) using the MTT assay and the caspase 3/7 activity was determined over the range of 62.5–1000 μg/mL.

**Results:**

Phytochemical analyses resulted in the characterization of 15 components, mainly flavonoids and lignans. The DCM extract showed significant antitrypanosomal activity (IC_50_: 36.21 μg/mL) and moderate activity against *Streptococcus pyogenes* (MIC value: 0.25 mg/mL). At a dose of 12.5 μg/mL, the DCM extract inhibited 73.6% of the plaque production by hepatitis A virus. CCRF-CEM cells were the most sensitive to both extracts (IC_50_: 12.5–22.7 μg/mL). The cytotoxicity was mediated by induction of apoptosis (19-fold increase in the cellular caspase 3/7 level after treatment with the DCM extract at 1 mg/mL).

**Conclusions:**

ME and DCM extract of *B. marginatum* showed anti-infective and antiproliferative effects.

## Introduction

Plants have been the foundation of traditional medicinal systems in many countries for hundreds of years to prevent or treat ailments and health disorders. Despite the tremendous success in manufacturing synthetic, semisynthetic therapeutic agents or both, approximately 80% of the world population still relies on herbal medicines for their health care [1].

The family Apiaceae comprises more than 3500 species, of which 300 are medicinally active and have been used in folk medicine [2]. Among the Apiaceae genera, *Bupleurum* encompasses about 180–190 species [3]. *Bupleurum marginatum* Wall. ex DC is a perennial herb of up to 60 cm in height, with oblanceolate leaves and yellow umbel flowers, and used in Chinese medicine [4].

*B. marginatum* has been widely used in Europe, China, Korea, and Japan in the form of herbal teas, either alone or in combination with other ingredients, for treatment of the common cold and inflammation [5,6]. In addition, it is employed to treat hepatitis, cancer, microbial infections, and fever associated with malaria [7-9].

The secondary metabolites of *B. marginatum* have been investigated previously, and various classes of compounds were identified. Specifically, saikosaponins were found in small amounts [10], while flavonoid glycosides and aglycones such as rutin, narcissin, isoquercetrin, isorhamnetin, and quercetin were commonly found in the plant [11]. In addition, lignans such as marginatoxin [11-13] and many phytosterols (stigmasterol, α-spinasterol, β-sitosterol, and daucosterol) have been described from both the aerial and subterranean parts [6,12].

Our previous work in 2009 [14] investigated the antibacterial, antifungal, antioxidant, anti-inflammatory and cytotoxic activity of the essential oil from the *B. marginatum* aerial parts. To our knowledge, the biological activities of different extracts from *B. marginatum* have not been studied in detail.

This study aims to investigate the antitrypanosomal, antimicrobial and antiviral activities of methanol (ME) and dichloromethane (DCM) extracts of *B. marginatum* aerial parts and the ability of both extracts to inhibit the growth of different cancer cell lines.

## Methods

### Plant materials

The aerial parts of *B. marginatum* were kindly provided by Prof. Thomas Efferth (University of Mainz, Mainz, Germany). The identity of the plant was ascertained by one of the authors (MA) in our laboratory by DNA barcoding and confirmed morphologically at the Botanical Garden, Heidelberg University (Heidelberg, Germany). Voucher specimens of the plant material were deposited at the Department of Biology, Institute of Pharmacy and Molecular Biotechnology, Heidelberg University under accession number IPMB P7367.

### Chemicals

Chemicals were purchased from AppliChem® (Darmstadt, Germany), Fluka® (Buchs, Switzerland), and Sigma® (Sternheim, Germany). The solvents used were of analytical grade unless otherwise mentioned, and were purchased from Merck® (Darmstadt, Germany), J.T. Backer® (Deventer, Netherlands), and Theo Seulberger® (Karlsruhe, Germany). Media and supplements for cell cultures were obtained from Gibco® (Invitrogen, Karlsruhe, Germany) and Greiner Labortechnik® (Frickenhausen, Germany).

### Preparation of ME and DCM extracts

The ME and DCM extracts were prepared by refluxing 100 g of finely milled, air-dried aerial parts of *B. marginatum* with 1.5 L of methanol or dichloromethane, respectively, for 6 h. The extracts were filtered, dried by passage over anhydrous sodium sulfate, and evaporated to dryness under reduced pressure at 40°C in a Büchi rotavapor R-200 with B-172 Büchi vacuum system (Büchi Labortechnik, Flawil, Switzerland). The dried extracts were stored at 4°C in the dark until analysis.

### General experimental procedures

#### LC-MS profiling of the extracts

The LC-ESI-MS system used consisted of a Merck Hitachi® HPLC system (Hitachi Ltd., Tokyo, Japan) composed of a binary L-6200A intelligent pump, an ERC-3215α degasser (ERC Inc., Saitama, Japan), a rheodyne injector with a 20-μl loop, and a LiChroCART RP-18, endcapped (5 μm), 250 × 4 mm i.d. column (Merck, Darmstadt, Germany) coupled with a Micromass VG Quattro II mass spectrometer (Waters, Manchester, United Kingdom). The ESI-MS system was operated using Waters MassLynx 4.0 software as described in our previous work [15]. The mobile phase was a linear gradient of 100% water (containing 0.5% formic acid) to 50% acetonitrile at a flow rate of 1 mL/min over 60 min, followed by a linear gradient to 100% acetonitrile over 10 min. Mass spectrometric detection of phenolics was performed in both the positive and negative ion modes over the range of 400–1400 *m/z* by N_2_ as a nebulizer gas and a source temperature of 120°C. The chromatograms were processed by Waters MassLynx® 4.0 software (Waters Corporation, Milford, MA , USA).

### Biological activities

#### Antitrypanosomal activity

Mature bloodstream forms of *Trypanosoma brucei brucei* TC221, the causative agent of Nagana (which can be used as a model for human sleeping sickness), were grown in Baltz medium supplemented with 20% inactivated fetal bovine serum and 1% penicillin-streptomycin. The cells were incubated in a humidified atmosphere containing 5% CO_2_ at 37°C.

Trypanocidal activity was determined by resazurin as a cell proliferation indicator dye as previously described [16]. The extracts were serially diluted with medium in a two-fold manner to attain final concentrations in the range of 250 to 3.9 μg/mL in 96-well Plates. *T. b. brucei* was seeded into 96-well plates at a density of 1 × 10^4^ cells/well in 100 μL of medium. The cells were then incubated with various concentrations (3.9–250 μg/ml) of the test samples for 24 h. Ten microliters of resazurin was added to each well and incubated for a further 24 h. The absorbance of the wells was recorded by a Tecan® plate reader (Männedorf, Switzerland) at dual wavelengths of 492 and 595 nm, and the IC_50_ was calculated [17].

Each sample concentration was tested in triplicate, and the experiments were repeated independently twice. The maximum concentration of the solvent (DMSO) did not exceed 1.25% in the medium that contained the highest concentration of each test sample. Diminazene aceturate was used as a positive control (IC_50_: 0.088 μg/mL) [18].

#### Antimicrobial activity

##### Microbial strains

The antimicrobial activity was evaluated by standard strains. The Gram-positive bacteria examined included *Bacillus subtilis* (ATCC 6051), *Staphylococcus aureus* (ATCC 29213), *Staphylococcus epidermidis* (ATCC 14990), *Streptococcus pyogenes* (ATCC 12344), *Streptococcus agalactiae* (ATCC 27956), and *Methicillin-Resistant Staphylococcus aureus* (MRSA; NTCC 10442). The Gram-negative bacteria *Escherichia coli* (ATCC 25922) and *Pseudomonas aeruginosa* (ATCC 27853) were also included, as well as the fungi *Candida albicans* (ATCC 90028) and *Candida glabrata* (ATCC MYA 2950). Multi-resistant clinical isolates from patients, such as *MRSA* 818014 and *MRSA* 818081, were also examined. All microorganisms were supplied by Medical Microbiology Laboratory, Hygiene Institute, Heidelberg University.

##### Inocula preparation

Prior to the test, bacterial and fungal cultures were prepared as follows. The bacterial cultures were subcultured in Columbia medium with 5% sheep blood (BD, Heidelberg, Germany) and incubated at 37°C for 24 h, while the fungal cultures were subcultured in CHROMagar® Candida (BD, Heidelberg, Germany) and incubated at 25°C for 48 h.

##### Agar diffusion method

Suspensions of microorganisms were prepared in a saline solution and adjusted with 0.5 McFarland Standard to a final concentration of approximately 1 × 10^6^ CFU/mL as previously reported [14]. Mueller Hinton agar (Biomerieux, Marcy l’Étoile, France) was inoculated with the pathogens. Wells with a diameter of 6 mm were cut out by a Pasteur pipette and loaded with 40 μL of 3.2 mg/mL extracts. DMSO, ampicillin, vancomycin, and nystatin were used as controls. The diameters of the growth inhibition zones were measured in triplicate after incubation at 37°C for 24 h (bacteria) or 48 h (fungi).

##### Determination of minimum inhibitory concentration (MIC) and minimum microbicidal concentration (MMC) values

The MIC was determined by the broth microdilution method [19]. Various sample concentrations (0.05–25 μg/mL) were dissolved in 5% DMSO and placed in a 96-well plate (Greiner Bio-one, Frickenhausen, Germany). The final count of the microbial suspension in Mueller Hinton broth (Fluka, Buchs, Switzerland) and Sabouraud Dextrose broth (Oxoid, Hampshire, UK) was adjusted to approximately 5 × 10^5^ CFU/mL for bacteria and fungi, respectively. The plates were incubated at 37°C for 24 h (bacteria) or 48 h (fungi)., The suspension (3 μL) from each incubated well was spread out on medium and incubated at 37°C for 24 h (bacteria) or 48 h (fungi) to determine the MMC. The MMC was defined as the lowest concentration of each extract that completely killed the microorganisms. Each test was performed in duplicate. The antibiotics ampicillin, vancomycin, and nystatin were used as positive controls.

#### Antiviral activity

Antiviral activity was determined by a plaque reduction assay [20]. Briefly, a confluent layer of Vero cells (CCL-81) was obtained by culturing the cells for 24 h in 0.5% CO_2_ at 37°C. The cells were inoculated, separately with herpes simplex virus type-1 (HSV-1), hepatitis A virus (HAV), or vesicular stomatitis virus (VSV) (1 × 10^-1^ ‒ 1 × 10^-7^ /ml) and incubated at 37°C for 1 h. The infected cell cultures (2 × 10^3^ PFU) were washed and overlaid with DMEM containing three concentrations of each extract (6.25, 12.5, and 50 ìg/mL) for 1 h at room temperature. Each concentration was tested in three replicates, and the cultures were overlaid with nutrient agarose (2 × DMEM/1.8% agarose [v/v]) containing 25 mM MgCl_2_. After 72 h of incubation, the cells were fixed with 10% formaldehyde in phosphate-buffer solution (pH 7.3) for 1 h and stained with 0.5% crystal violet in 20% ethanol. The plaques were counted, and the percentage of viral inhibition was calculated as [1 - fV*d*/V*c*)] × 100, where V*d* and V*c* are the numbers of plaques in the presence and absence of the test samples, respectively. Acyclovir was employed as a positive control.

### Cytotoxicity

#### Cell cultures

Human MCF-7 (breast cancer), PANC-1 (pancreatic carcinoma), MIA PaCa-2 (pancreatic cancer), Caco-2 (colon cancer), and African green monkey kidney COS-7 (fibroblast-like epithelium) and CCL-81 (Vero) cell lines were maintained in DMEM complete medium (L-glutamine supplemented with 10% heat-inactivated fetal bovine serum, 100 U/mL penicillin, and 100 U/mL streptomycin), with addition of 1 mM sodium pyruvate and 10 mM non-essential amino acids for culture of Caco-2 cells. Human CCRF-CEM (leukemia) and HL-60 (myeloid) cells were maintained in RPMI complete medium. The cells were maintained at 37°C in a humidified atmosphere containing 5% CO_2_. All experiments were performed with cells in the logarithmic growth phase.

#### Cytotoxicity and cell proliferation assay

Cytotoxicity was determined in triplicate using the MTT cell viability assay [21]. Exponentially growing cells of each cell line were seeded in 96-well plates (Greiner Labortechnik®, Frickenhausen, Germany) at 2 × 10^4^ cells/well. The cells were cultured for 24 h, and then incubated with various concentrations of the extracts ranging from 1 to 1000 μg/mL at 37°C for 24 h. Subsequently, the cells were incubated with 0.5 mg/mL MTT for 4 h. The formed formazan crystals were dissolved in 200 μL of DMSO. The absorbance was detected at 570 nm by a Tecan® Safire II Reader (Männedorf, Switzerland). The cell viability (percentage) of three independent experiments was calculated as follows:

$$\begin{array}{lll} \text{Cell}\;\text{viability}\;\left(\%\right) & = & \left(\text{OD}\;\text{of}\;\text{treated}\;\text{cells}\right)/\\ & & \left(\text{OD}\;\text{of}\;\text{control}\;\text{cells}\right)\times 100\%. \end{array}$$

The cytotoxic agent doxorubicin was used as a positive control.

#### Apoptosis assay

The Caspase-Glo™ 3/7 Assay (Promega®, Mannheim, Germany) was used to detect caspase 3/7 activities in MIA PaCa-2 cells after treatment with each type of extract (62.5 to 1000 μg/mL). This test provides a proluminescent caspase 3/7 substrate, which contains the caspase 3-specific tetrapeptide sequence DEVD in a reagent mix, and is optimized for cell lysis and determination of caspase activities. Cells cultured in DMEM were seeded in 96-well plates and treated with the extracts (62.5 μg to 1 mg). Subsequently, 100 μL of caspase 3/7 reagent was added to the wells after 6 or 24 h of treatment, mixed, and incubated at room temperature for 30 min [22]. The luminescence was measured by a Mithras LB 940 instrument (Berthold Technologies, Bad Wildbad, Germany). Cellular apoptosis was expressed as the fold change relative to the untreated control in three independent experiments.

### Data analysis

All biological experiments were repeated at least three times. Data were presented as the mean ± standard deviation (SD). The IC_50_ was determined as the drug concentration that resulted in a 50% reduction in cell viability or inhibition of biological activity. The IC_50_ values were calculated by a four parameter logistic curve (SigmaPlot® 11.0, Systat Software Inc., San Jose CA, USA). The significance of differences in data between the groups was determined by one-way analysis of variance followed by the Tukey test for equality of variances using SPSS® 11.0 (SPSS Inc., Chicago, USA). Differences were considered statistically significant at *P* < 0.05.

## Results and discussion

### HPLC profiling and chemical compositions of the extracts

**Figure 1:**
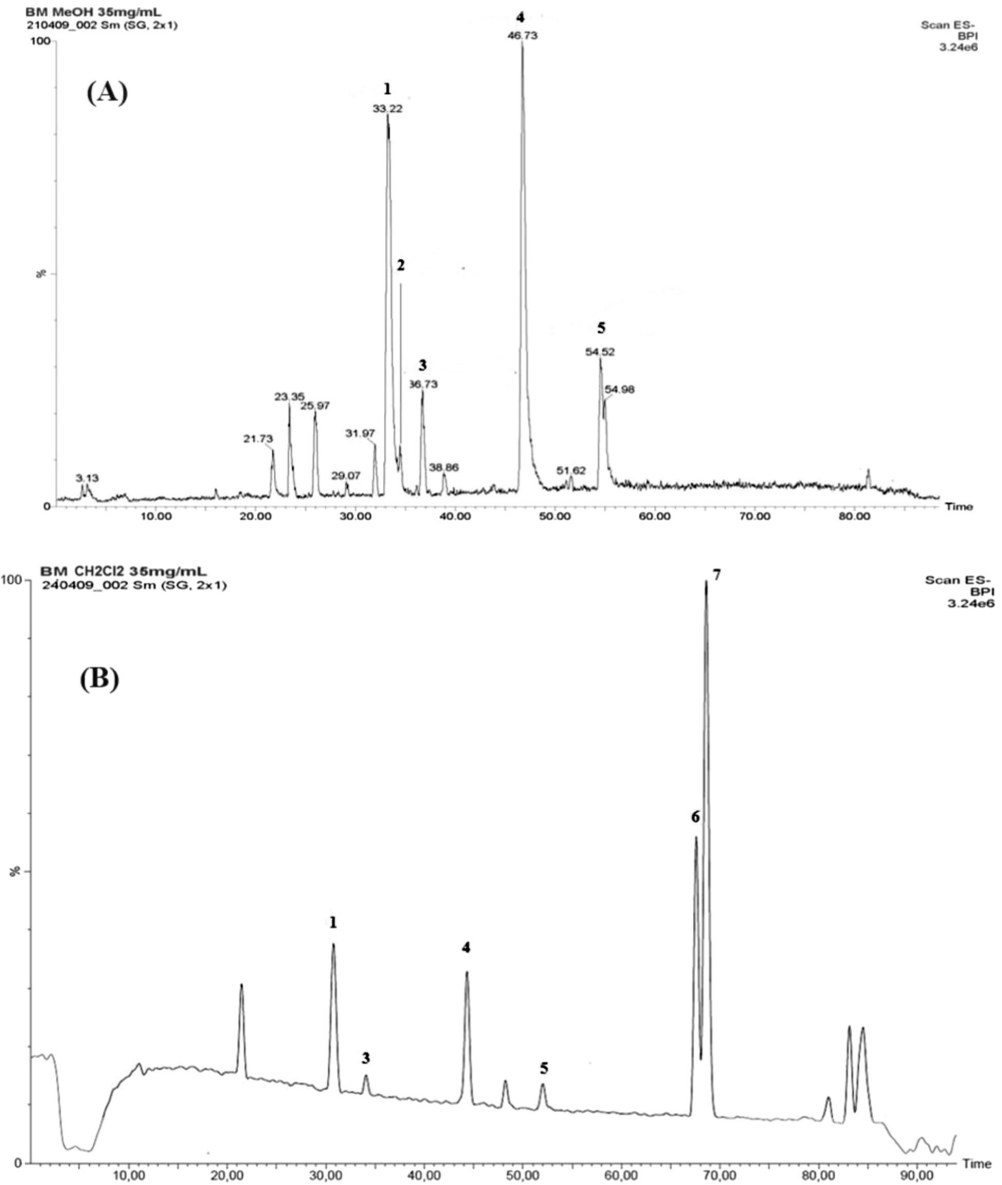
**HPLC-MS profiling of the ME (A) and DCM (B) extracts from the aerial part of *B. marginatum*.** 1: rutin; 2: narcissin; 3: isoquercitrin; 4: quercetin; 5: isorhamnetin; 6: lignan 2; 7: marginatoxin.

The chromatographic profiles were established by HPLC/MS (Figure 1). The ME extract (Figure 1A) revealed the presence of high contents of flavonoids, and to lesser extents lignans, sterols, and triterpenoid saponins, as previously reported by the authors [11]. The DCM extract (Figure 1B) showed a similar pattern of secondary metabolites with higher yields of lignans and terpenoids compared with those detected in the ME extract. These results are in accordance with our previously detected terpenoids in the *n*-hexane extract [14].

**Figure 2:**
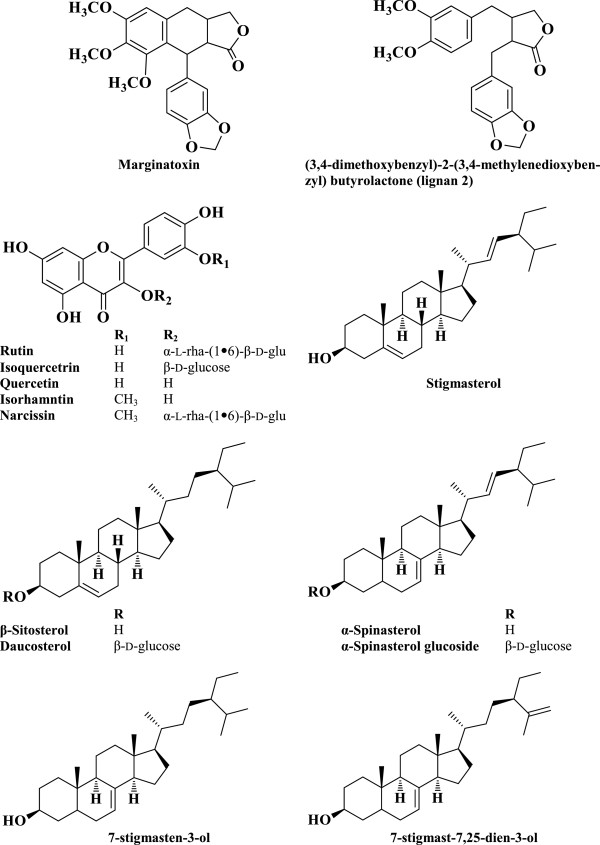
**Structures of the isolated compounds from the aerial part of *B. marginatum*.**

In the *B. marginatum* ME and DCM extracts, the following compounds (Figure 2) were identified unequivocally: quercetin and its glycosides rutin and isoquercetrin; isorhamnetin and its glycoside narcissin; (3,4-dimethoxybenzyl)-2-(3,4-methylenedioxybenzyl) butyrolactone; and marginatoxin. In addition, sterols such as β-sitosterol and α-spinasterol and their glucosides, daucosterol, stigmasterol, 7-stigmasten-3-ol, 7-stigmast-7,25-dien-3-ol, and hexadecanol were either isolated or identified from the different extracts.

### Activity against *T. b. brucei*

**Figure 3:**
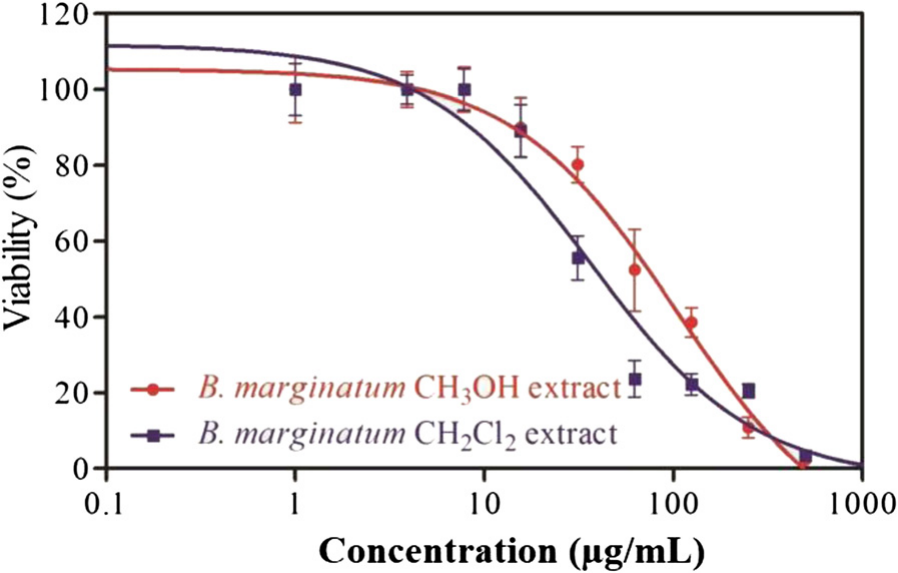
**Dose-dependent growth inhibition of *T. b. brucei *** **(resazurin assay).**

The trypanocidal activities of both extracts were illustrated in Figure 3. The ME and DCM extracts showed moderate activities with IC_50_ values of 104.80 ± 5.11 (*P* = 0.0027) and 36.21 ± 2.68 (*P* = 0.0014) μg/mL, respectively. The selectivity indexes of the two extracts were calculated as 2.3 and 2.9, respectively, indicating low selectivity against trypanosomes compared with their cytotoxicity toward HL-60 cells.

Based on previous work [23,24], many flavonoid aglycones exhibitantitrypanosomal activity in both *in vitro* and *in vivo* models. However, the activity of most flavonoids against *T. b. brucei* and the selectivity of these compounds were related to the presence of a free phenolic OH group in position 6 which can dissociate to a phenolate ion under physiological conditions [23]. Thus, it is probable that the activity of the extracts could be related to the presence of several flavonoids that could interact together in synergistic or even additive manners [25].

To our knowledge, few studies have addressed the antitrypanosomal activity of lignans [26]. However, two closely structurally related lignans, justicidin B and piscatorin, which were isolated from the aerial parts of *Phyllanthus piscatorum* Kunth (Euphorbiaceae), exhibited strong activity against the bloodstream forms of *Trypanosoma brucei rhodesiense* with IC_50_ values of 0.55 and 6.1 μM, respectively [27].

### Antimicrobial activity

The antimicrobial activity of the ME and DCM extracts was assessed by the agar diffusion and microdilution methods, and the MIC and MMC values were determined (Tables 1 and 2). In general, both extracts showed growth inhibition of standard Gram-positive and Gram-negative bacteria in the diffusion assay. However, no inhibition zones could be detected for the two fungi and the resistant pathogenic isolates.

**Table 1:** **Mean inhibition zones of the *B. marginatum *** **extracts against different pathogens determined by the agar diffusion method**

**Microorganisms**	**Diameter of inhibition zone (mm)**				
	**CH**_**3**_**OH extract 640 μg/well**	**CH**_**2**_**Cl**_**2 **_**extract 640 μg/well**	**Ampicillin 10 μg/well**	**Vancomycin 10 μg/well**	**Nystatin 10 μg/well**
**Gram positive**					
MRSA 818014	NI	NI	5.3 ± 0.3	10.3 ± 0.7	NT
MRSA 818081	NI	NI	7.2 ± 0.0	10.6 ± 1.3	NT
MRSA NCTC 10442	NI	NI	NI	10.0 ± 0.2	NT
*Staphylococcus aureus* ATCC 29213	4.3 ± 0.7 (P*^1,2^ = 0.001, 0.008)	5.1 ± 1.4 (*P*^*1,2*^ = 0.001, 0.008)	24.0 ± 1.5	10.7 ± 1.6	NT
*Staphylococcus epidermidis* ATCC 14990	4.3 ± 2.5 (P*^1,2^ = 0.016, 0.027)	5.6 ± 1.3 (*P*^*1,2*^ = 0.021, 0.019)	12.2 ± 1.7	10.0 ± 0.5	NT
*Streptococcus pyogenes ATCC 12344*	5.7 ± 1.6 (P*^1,2^ = 0.006, 0.021)	7.2 ± 2.1 (*P*^*1,2*^ = 0.011, 0.017)	25.0 ± 1.2	15.3 ± 1.6	NT
*Streptococcus agalactiae* ATCC 27956	5.2 ± 1.1 (P*^1,2^ = 0.024, 0.021)	5.2 ± 1.5 (*P*^*1,2*^ = 0.023, 0.021)	17.1 ± 1.5	12.0 ± 1.7	NT
*Bacillus subtilis* ATCC 6051	NI	4.2 ± 2.1 (*P*^*1,2*^ = 0.012, 0.038)	12.7 ± 0.5	8.7 ± 1.2	NT
**Gram negative**					
*Pseudomonas aeruginosa* ATCC 27853	4.1 ± 0.0	5.3 ± 1.3	NI	NI	NT
*Escherichia coli* ATCC 25922	4.3 ± 0.2	NI	5.4 ± 1.2	NI	NT
**Fungi**					
*Candida albicans* ATCC 90028	NI	NI	NT	NT	10.0 ± 1.2
*Candida glabrata* ATCC MYA 2950	NI	NI	NT	NT	12.1 ± 1.2

Data are presented as means ± SD (n = 3).

NI: no inhibition zone; NT: not tested.

*P*^*1,2*^: Significant differences relative to ampicillin and vancomycin, respectively.

**Table 2:** **Minimum inhibitory concentrations (MICs) and minimum microbicidal concentrations (MMCs) of the *B. marginatum *** **extracts against different pathogens determined by the broth microdilution method**

**Microorganisms**	**CH**_**3**_**OH extract mg/mL**		**CH**_**2**_**Cl**_**2 **_**extract mg/mL**		**Ampicillin μg/mL**		**Vancomycin μg/mL**		**Nystatin μg/mL**	
	**MIC**	**MMC**	**MIC**	**MMC**	**MIC**	**MMC**	**MIC**	**MMC**	**MIC**	**MMC**
**Gram positive**										
MRSA 818014	4	> 4	4	> 4	6.2	6.2	0.8	0.8	NT	NT
MRSA 818081	4	> 4	4	> 4	6.2	50	0.8	0.8	NT	NT
MRSA NCTC 10442	4	> 4	4	> 4	6.2	12.5	0.8	1.6	NT	NT
*Staphylococcus aureus* ATCC 29213	4	> 4	4	> 4	0.4	3.1	0.4	0.8	NT	NT
*Staphylococcus epidermidis* ATCC 14990	2	> 4	2	> 4	0.4	0.8	0.8	1.6	NT	NT
*Streptococcus pyogenes ATCC 12344*	0.5	4	0.25	0.75	0.1	0.1	0.1	0.2	NT	NT
*Streptococcus agalactiae* ATCC 27956	0.5	4	0.5	4	0.1	0.2	0.4	0.4	NT	NT
*Bacillus subtilis* ATCC 6051	0.5	2	0.5	1	0.1	0.8	0.2	0.8	NT	NT
**Gram negative**										
*Pseudomonas aeruginosa* ATCC 27853	4	> 4	4	> 4	NI	NI	NI	NI	NT	NT
*Escherichia coli* ATCC 25922	2	4	4	> 4	6.2	12.5	NI	NI	NT	NT
**Fungi**										
*Candida albicans* ATCC 90028	NI	NT	NI	NT	NT	NT	NT	NT	1.6	1.6
*Candida glabrata* ATCC MYA 2950	NI	NT	NI	NT	NT	NT	NT	NT	1.6	1.6

NI: no inhibition; NT: not tested.

All of the tested Gram-positive bacteria were susceptible to the ME and DCM extracts with MIC values ranging from 0.25 to 4 mg/mL. *S. agalactiae* and *B. subtilis* were sensitive to both extracts with similar MIC values of 0.5 mg/mL. *S. pyogenes* was the most susceptible to the DCM extract with an MIC value of 0.25 mg/mL. The Gram-negative pathogens and fungi were less sensitive, and their MIC values were ≥ 2 mg/mL. The two extracts did not differ significantly in their antimicrobial actions for all of the susceptible microorganisms. However, they were significantly different from both ampicillin and vancomycin as seen from the difference in inhibition zones in Table 1.

Although it was expected that the ME extract of *B. marginatum* would possess superior activity compared with the DCM extract based on the richness and diversity of secondary metabolites, our findings did not reveal a significant difference. The expected higher activity of the ME extract was related to the presence of saikosaponins, which function as detergents and mostly exist in their mono-desmosidic forms [28]. These compounds can intercalate *via* their lipophilic moiety with the lipophilic membrane bilayer of the pathogens after forming a complex with cholesterol, while the hydrophilic sugar part remains outside of the cell and interacts with glycoproteins or glycolipids [28]. A loss of the membrane integrity allows other highly polar flavonoid glycosides to penetrate the pathogen and exert their effects. On this basis, the antimicrobial activity is likely to be influenced by the synergistic and additive effects of all components [28].

### Antiviral activity

The ME and DCM extracts showed promising antiviral activity at a dose of 50 μg/mL. HAV was the most sensitive, showing 76.6% and 81.4% inhibition of viral replication with the ME and DCM extracts, respectively (Table 3). The antiviral activity of the different *Bupleurum* species have been related to the contents of saikosaponins, which exert powerful and selective antiviral activity [29-31]. However, the DCM extract with a low steroid content showed a higher activity than the ME extract against the three viruses, which might be attributed to its high contents of lignans and flavonoid aglycones.

**Table 3:** **Effects of the *B. marginatum *** **extracts on viral replication using direct plaque reduction assays**

	**% inhibition in viral replication**		
	**HSV-1**	**HAV**	**VSV**
**CH**_**3**_**OH extract**			
6.25 μg/mL	21.75 ± 1.18	33.71 ± 1.82	12.87 ± 1.88
12.50 μg/mL	56.44 ± 1.60	65.01 ± 2.12	41.37 ± 2.16
50.00 μg/mL	66.26 ± 2.72 (P* = 0.0011)^a^	76.56 ± 4.29 (P* = 0.025)^b^	56.59 ± 2.03 (P* = 0.0024)^c^
**CH**_**2**_**Cl**_**2 **_**extract**			
6.25 μg/mL	29.45 ± 1.23	42.51 ± 2.21	39.27 ± 1.95
12.50 μg/mL	59.53 ± 3.41	73.67 ± 3.03	69.55 ± 3.16
50.00 μg/mL	73.91 ± 4.13 (P* = 0.0017)^a^	81.42 ± 6.15 (P* = 0.019)^b^	79.96 ± 4.51 (P* = 0.0009)^c^

Data are presented as means ± SD (n = 3).

HSV-1: herpes simplex virus type-1; HAV: hepatitis A virus; VSV: vesicular stomatitis virus.

P*: Significant difference relative to acyclovir.

^a^Significant difference at *P* = 0.048.

^b^Significant different at *P* = 0.037.

^c^Significant difference at *P* = 0.001.

Several modes of cytotoxic and antiviral activity were associated with lignans, including tubulin binding, reverse transcriptase, and topoisomerase inhibition [32,33]. However, the most relevant mechanism associated with lignans involved binding to tubulin, disruption of the cellular cytoskeleton and spindle apparatus of the infected host cells, and interfering with some critical viral processes [32]. Marginatoxin, isolated from a *B. marginatum* extract, showed high structural similarity with podophyllotoxin, which was previously reported to show potent activity against HSV-1, measles virus, VSV, and many other viruses [34-36].

In addition, flavonoids constitute the largest group of secondary metabolites with antiviral activity in the entire plant kingdom. The several phenolic groups in the flavonoid skeleton, which can dissociate into phenolate ions and form hydrogen and ionic bonds with amino acid residues of proteins, facilitates the antiviral activity by inhibiting viral polymerase activity and binding with viral nucleic acid or viral capsid proteins, leading to a reduction in viral replication [37,38]. Most flavonoids, including quercetin and rutin, show promising activity against HSV-I, HIV-1, HIV-2, poliovirus type 1, parainfluenza virus type 3, and respiratory syncytial virus [39-41].

### Cytotoxicity and apoptosis

**Figure 4:**
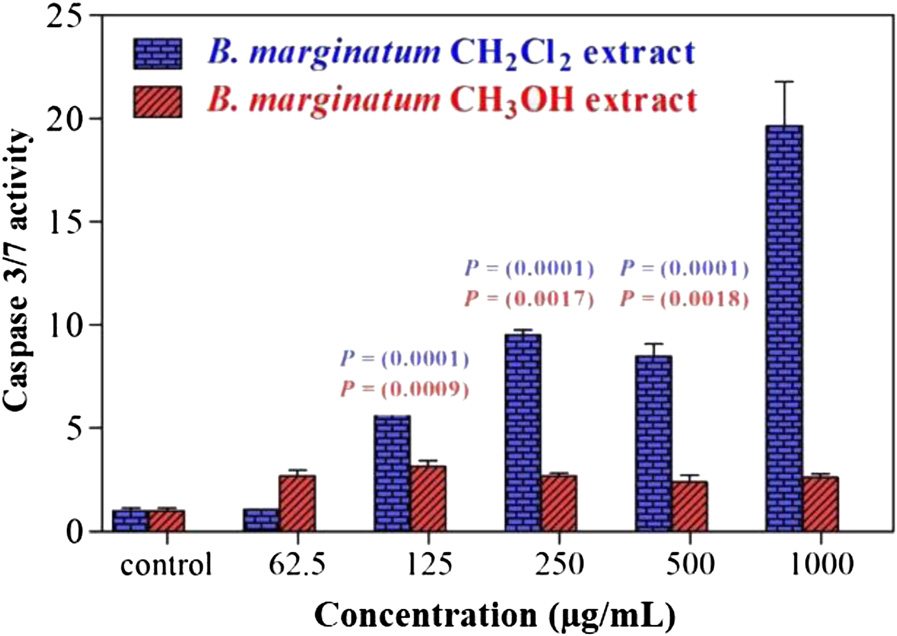
**Induction of apoptosis in MIA PaCa-2 cells by *B. marginatum *extracts.** Caspase 3/7 activation was measured after 24 h of treatment.

The cytotoxicity of the ME and DCM extracts was evaluated by eight different human cancer cell lines, Caco-2, CCL-81, CCRF-CEM, COS-7, HL-60, MCF-7, MIA PaCa-2, and PANC-1, after 24 h of incubation. The IC_50_ values are shown in Table 4. The cytotoxicity of the ME extract ranged from 22.5 to 576.0 μg/mL, while the DCM extract was more cytotoxic and had IC_50_ values ranging from 12.5 to 72.8 μg/mL. Both CCRF-CEM and MIA PaCa-2 cells were highly sensitive to both extracts, while the green monkey cells were the most resistant cell lines. Caspase 3/7 activity was used to measure the ability of the extracts to initiate the apoptotic cascade. Both extracts activated caspase 3/7. The increase was most apparent with the DCM extract, for which a 1 mg/mL dose produced 19-fold stimulation (Figure 4). The tested extracts showed higher selectivity for cancer cells when compared with normal hepatocytes. The ME extract had no cytotoxic activity against normal human cells over the range of 1–1000 mg/mL (unpublished data).

**Table 4:** **Cytotoxicities of the *B. marginatum *** **extracts (IC**_**50 **_**values; μg/ml) after 24 h of incubation**

**Cell lines**	**Methanol extract (μg/mL)**	**Dichloromethane extract (μg/mL)**	***P***^**1 **^**value**
**Caco-2**	108.44 ± 11.43 (P* = 0.012)	56.81 ± 6.30 (P* = 0.009)	0.0011
**CCRF-CEM**	22.75 ± 2.76 (P* = 0.014)	12.54 ± 1.87 (P* = 0.004)	0.0335
**CCL-81**	307.51 ± 13.76 (P* = 0.007)	72.81 ± 6.15 (P* = 0.013)	0.0016
**COS-7**	576.0 ± 11.65 (P* = 0.009)	67.40 ± 2.76 (P* = 0.021)	0.0019
**HL-60**	100.88 ± 4.02 (P* = 0.021)	35.07 ± 2.28 (p* = 0.007)	0.0026
**MIA PaCa-2**	22.51 ± 2.98 (P* = 0.023)	14.98 ± 1.09 (P* = 0.018)	0.0388
**MCF-7**	91.18 ± 8.99 (P* = 0.011)	51.00 ± 5.20 (P* = 0.041)	0.0279
**PANC-1**	34.81 ± 3.01 (P* = 0.031)	27.62 ± 2.96 (P* = 0.022)	0.0281

Data are presented as means ± SD (n = 3).

P*: Significant difference relative to doxorubicin.

*P*^1^: Significant difference relative to each other.

Several other *Bupleurum* species showed similar cytotoxicity against different cell lines [42-44]. The mechanism of this cytotoxicity was attributed to the saikosaponin content, especially the saponins with an epoxy bridge [42]. Cytotoxicity is mainly mediated through cell cycle arrest in the G_2_/M phase or inhibition of tubulin polymerization [45]. In addition, induction of apoptosis *via* the activation of kinases 1/2 and several caspases might be involved [43-45].

Furthermore, quercetin and lignans contributed to the overall cytotoxicity of both extracts. Quercetin blocks signal transduction pathways by inhibiting many kinases, including protein tyrosine kinases and serine/threonine protein kinases, resulting in arrest of the cell cycle at the late G_1_ phase [46] with a proapoptotic activity through activation of KRAS and PI3K [47]. Most lignans, especially those with a structural similarity to podophyllotoxin are known cytotoxic agents [48]. These agents bind to tubulin, leading to inhibition of microtubule formation, and inhibit DNA topoisomerase, thus blocking cell division in the late G_2_[49].

The low cytotoxicity of both extracts toward Caco-2 cells compared with the other tested cancer cell lines may arise through the overexpression of multidrug-resistance genes, such as MDR1 and MRP1, in this cell line [50,51]. These ATP-dependent efflux transporters facilitated the efflux of xenobiotics from Caco-2 cells, thereby reducing th\e internal concentration of cytotoxic drugs and thus their activity [52].

In this work, we investigated the antimicrobial activity of *Bupleurum* extracts, especially against Gram-positive bacteria such as *Streptococcus* and *Bacillus.* Moreover, the antitrypanosomal and antiviral activities of the DCM extract were explored against three different viruses. Further studies of the isolated compounds present in the extracts, particularly lignans, which might serve as lead compounds and could be modified to increase their selectivity were required. However, clinical reports should be collected from patients using these *Bupleurum* preparations to monitor the efficacy, as well as any side effects or herbal drug interactions.

## Conclusions

In this study, ME and DCM extracts of *B. marginatum* showed anti-infective and antiproliferative effects.

## Competing interests

The authors declare that they have no competing interests.

## Authors’ contributions

MLA and MW designed the study. MLA, MZL, RH, SYE, SHE, EN, FH, MY, AT and DKperformed the experiments. MLA and MW wrote the manuscript. All authors read and approved the final manuscript.
